# Supplementary material for: Hydrogenation versus hydrogenolysis during alkaline electrochemical valorization of 5-hydroxymethylfurfural over oxide-derived Cu-bimetallics
Source: Nat Commun. 2023 Aug 5;14:4708. doi: 10.1038/s41467-023-40463-y (PMC10404266; doi:10.1038/s41467-023-40463-y)
Supplement: Supplementary file 1 — Supplementary Information [file 41467_2023_40463_MOESM1_ESM.pdf]

SI- Hydrogenation versus Hydrogenolysis during Alkaline Electrochemical Valorization of 5-Hydroxymethylfurfural over oxide-derived Cu-bimetallics

Philipp Hauke[1], Thomas Merzdorf [1], Malte Klingenhof [1] and Peter Strasser[1]

1 The Electrochemical Energy, Catalysis, and Materials Science Laboratory, Department of Chemistry, Chemical Engineering Division, Technical University Berlin, Berlin, Germany

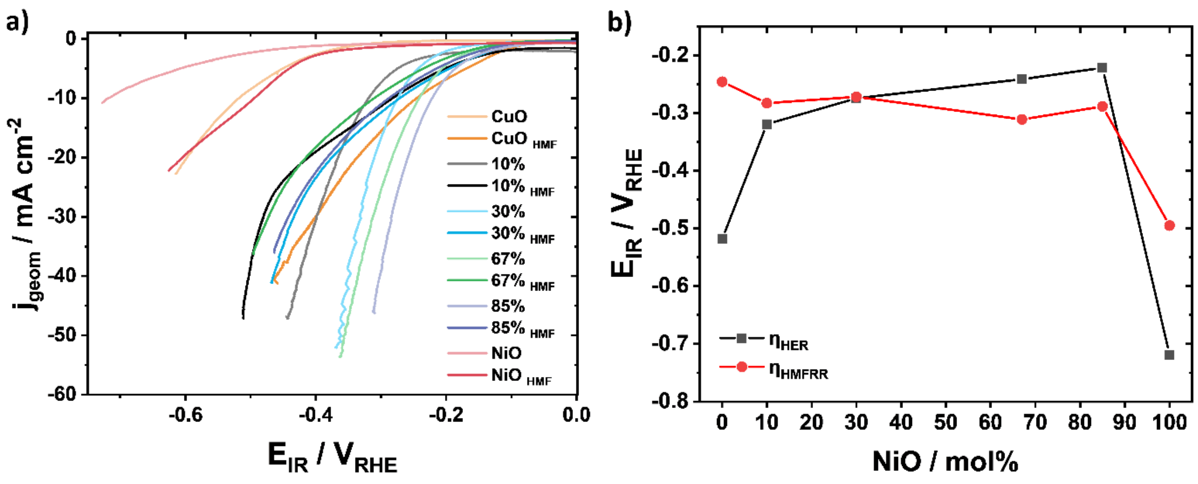

Supplementary Fig. 1: RDE measurements of CuO/NiO comparing the activity of different metal oxide molar ratios (mol%). The Cu to Ni ratio was varied by different Ni precursor amounts. a) LSV measurements were taken between 0 V_{RHE} to -0,8 V_{RHE} at a scan rate of 10 mV s^{-1} in 0.1 M KOH with and without 10 mM 5-HMF. All measurements are internal resistance (IR) corrected. b) resulting overpotentials from a) for HMFRR (red) and HER (black) at 10 mA cm^{-2} for different NiO mol%.

Supplementary Discussion I

To evaluate a preferred molar ratio of CuO and NiO, we tracked the HMF reduction reaction (HMFRR) activity and the HER activity at 10 mA cm^{-2}. The HER activity is assumed to be a measure for H_{ad} coverage and chemisorption under reactive conditions. Chemisorbed H_{ad} enables desired Langmuir-Hinshelwood-type multi-electron HMFRR, yet also leads to competitive, undesired Volmer-Tafel Hydrogen Evolution. Based on this balance, we targeted a 10 mol% metal Ni/Cu ratio in the two-phase catalysts due to its most favorable HMFRR activity coupled to a balanced HER activity. In order to maintain comparability, the molar ratio of 10% was kept for other catalysts. We are aware that, for the sake of the present study, the optimum ratio may vary slightly for Co and Fe based bimetallic oxides. However, as shown in Supplementary Fig. 1b, the HER and HMFRR reactivity were similar over most of the molar range.

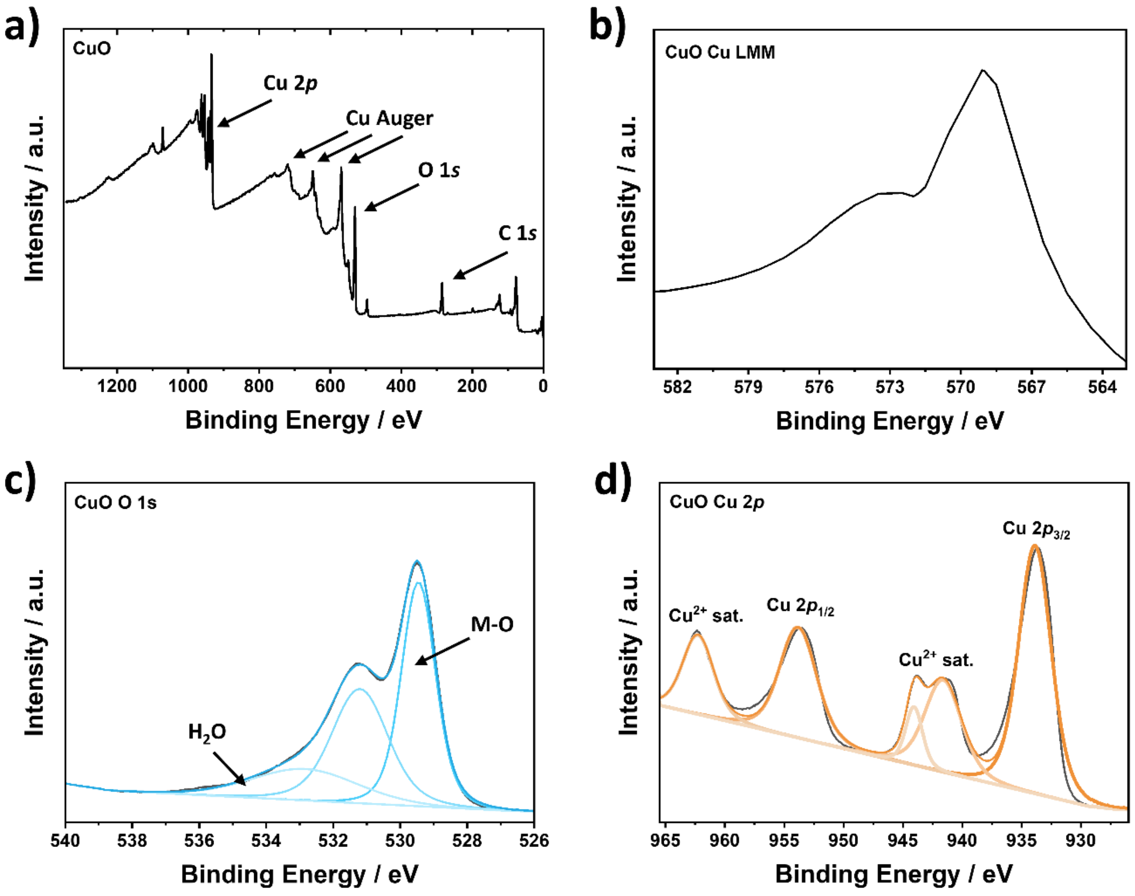

Supplementary Fig. 2: XPS measurements of CuO powder. a) Survey spectrum, b) Cu LMM Auger range c) O 1s region, d) Cu 2p region. Measurements are given in black whereas Casa XPS fits are given in light blue for O and orange for Cu.

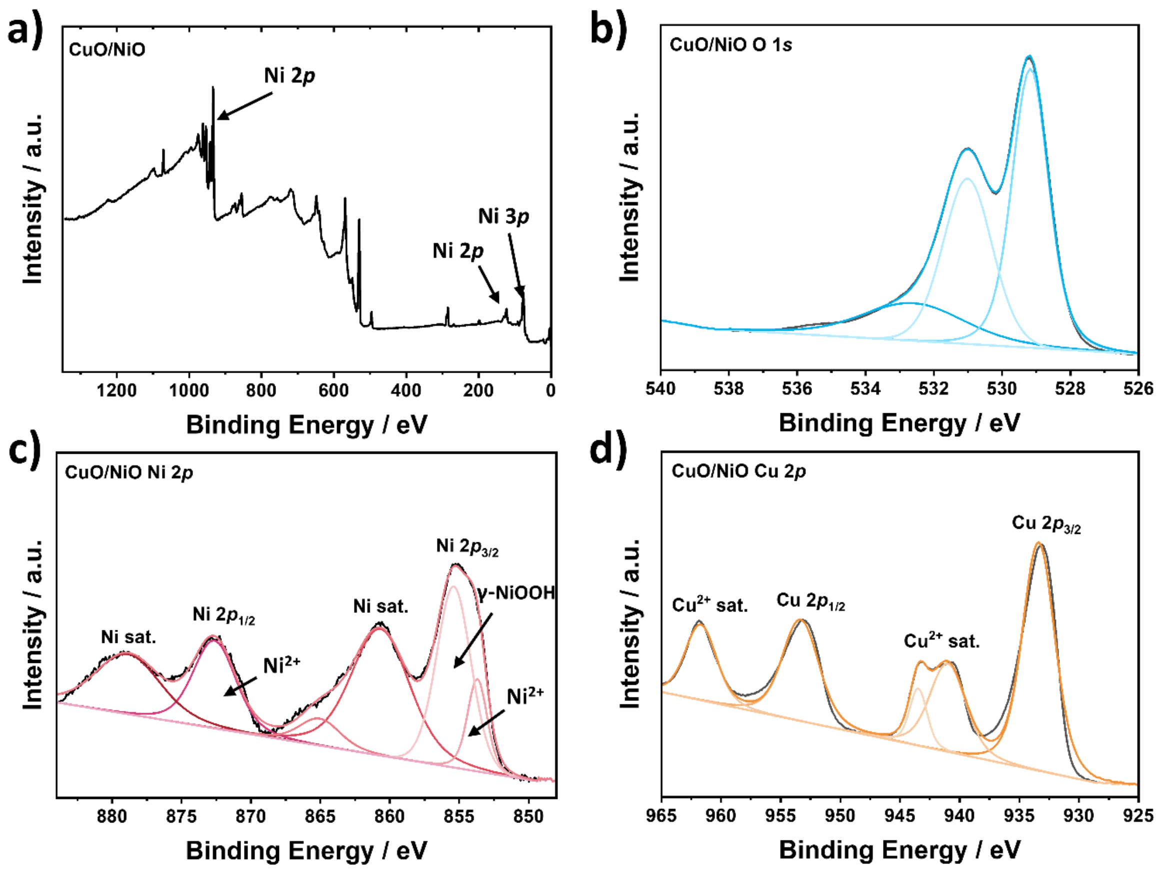

Supplementary Fig. 3: XPS measurements of CuO/NiO powder. a) Survey spectrum, b) O 1s region, c) Cu 2p region, d) Ni 2p region. Measurements are given in black whereas Casa XPS fits are given in yellow for C, light blue for O, orange for Cu and red for Ni.

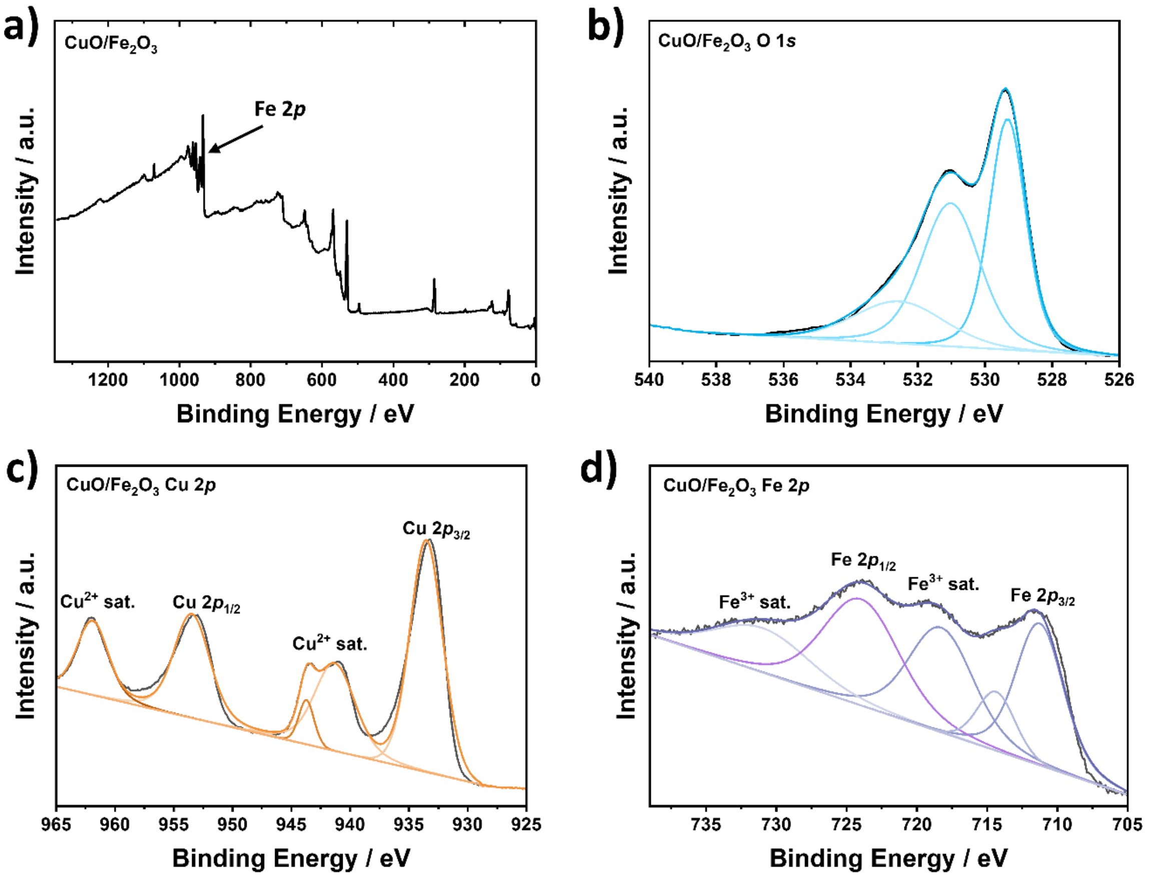

Supplementary Fig. 4*:* XPS measurements of CuO/Fe_2O_3 powder. a) Survey spectrum, b) O 1s region, c) Cu 2p region, d) Fe 2p region. Measurements are given in black whereas Casa XPS fits are given in yellow for C, light blue for O, orange for Cu and purple for Fe.

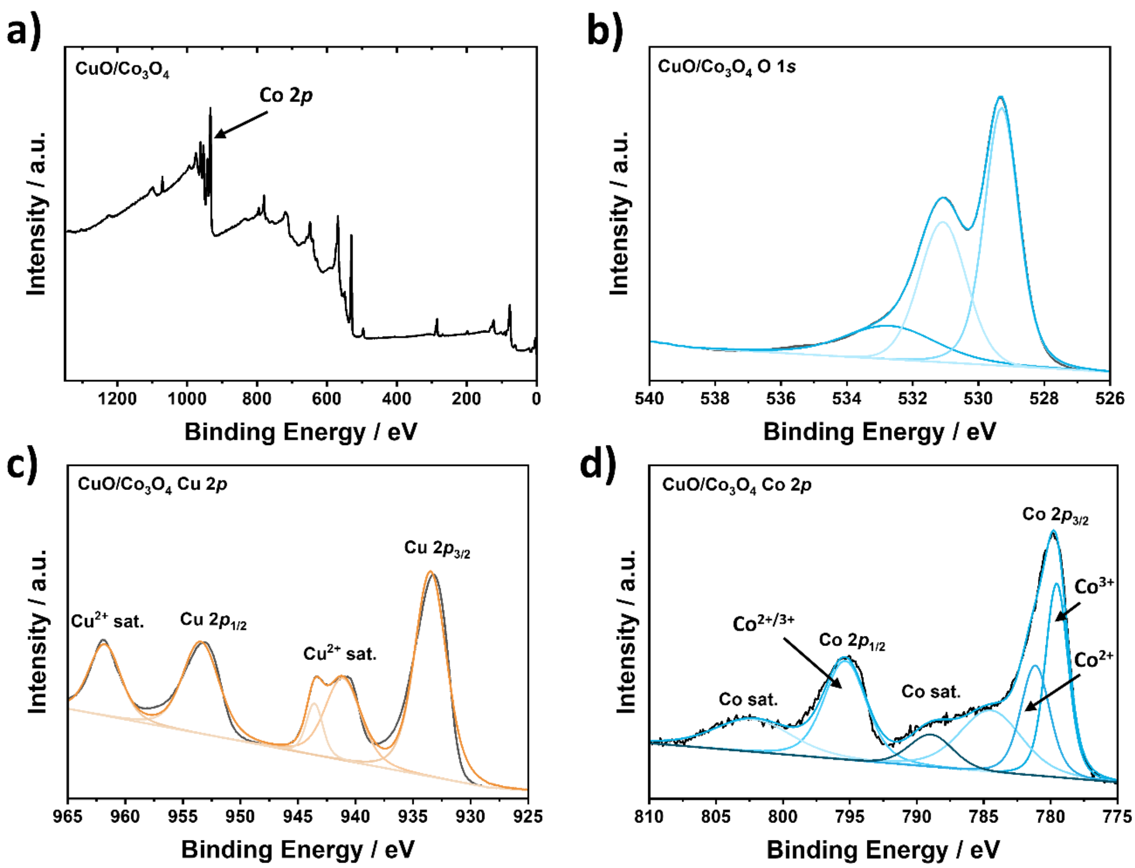

Supplementary Fig. 5: XPS measurements of CuO/Co_3O_4 powder. a) Survey spectrum, b) O 1s region, c) Cu 2p region, d) Co 2p region. Measurements are given in black whereas Casa XPS fits are given in yellow for C, light blue for O, orange for Cu and blue for Co.

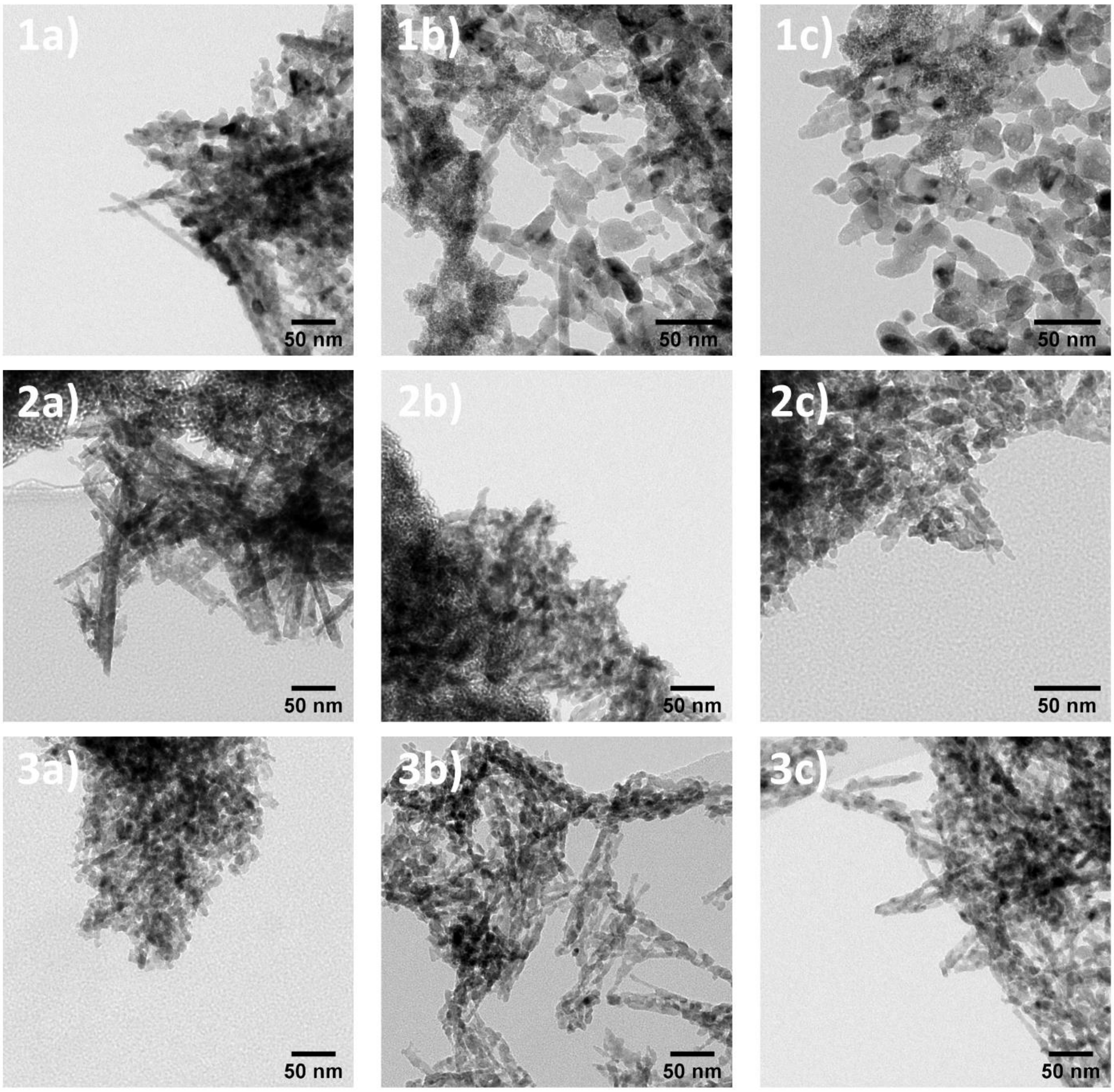

Supplementary Fig. 6: TEM images of the powder CuO/MO$_x$ catalysts. 1a)-c) showing CuO/NiO, 2a)-c) showing CuO/Fe_2O_3 and Figure 3a)-c) showing CuO/Co_3O_4. A scale is provided in the right bottom corner.

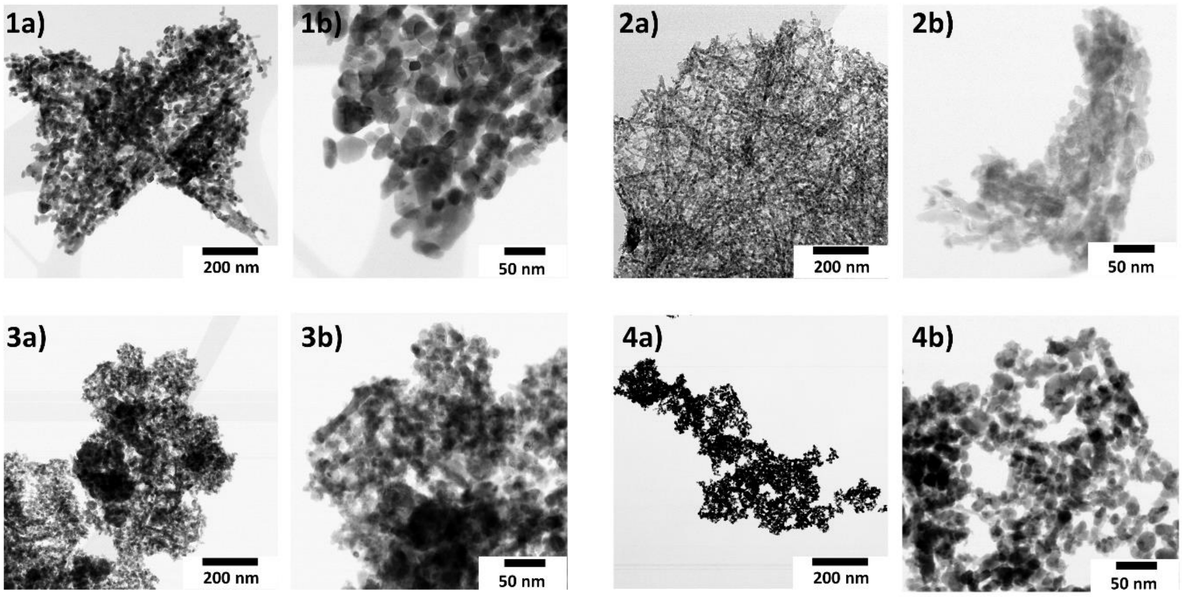

Supplementary Fig. 7: HR-STEM images of the powder CuO and CuO/MO_x catalysts. 1a) and b) showing CuO, 2a) and b) showing CuO/NiO, 3a) and b) showing CuO/Fe_2O_3 and Figure 4a) and b) showing CuO/Co_3O_4. A scale is provided in the right bottom corner.

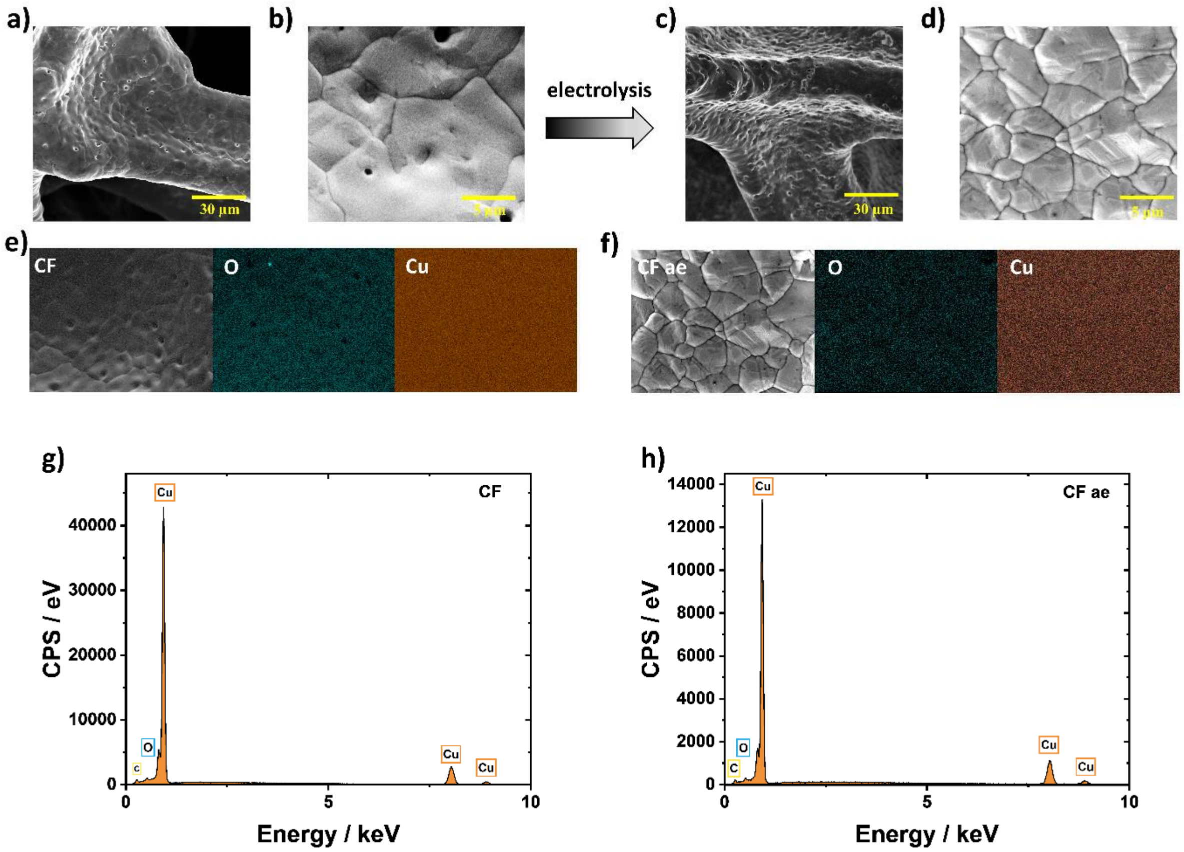

Supplementary Fig. 8: SEM-EDX measurements of CF before and after electrolysis. a) and b) SEM images of CF before electrolysis at 1000x and 5000x magnification. c) and d) SEM images of CF after electrolysis (ae) at 1000x and 5000x magnification. e) SEM EDX images before electrolysis of CF with O in torques and Cu in orange. f) SEM EDX images after electrolysis of CF with O in torques and Cu in orange. g) SEM EDX results before electrolysis of CF with O in torques, C in yellow and Cu in orange. h) SEM EDX results after electrolysis of CF with O in torques, C in yellow and Cu in orange.

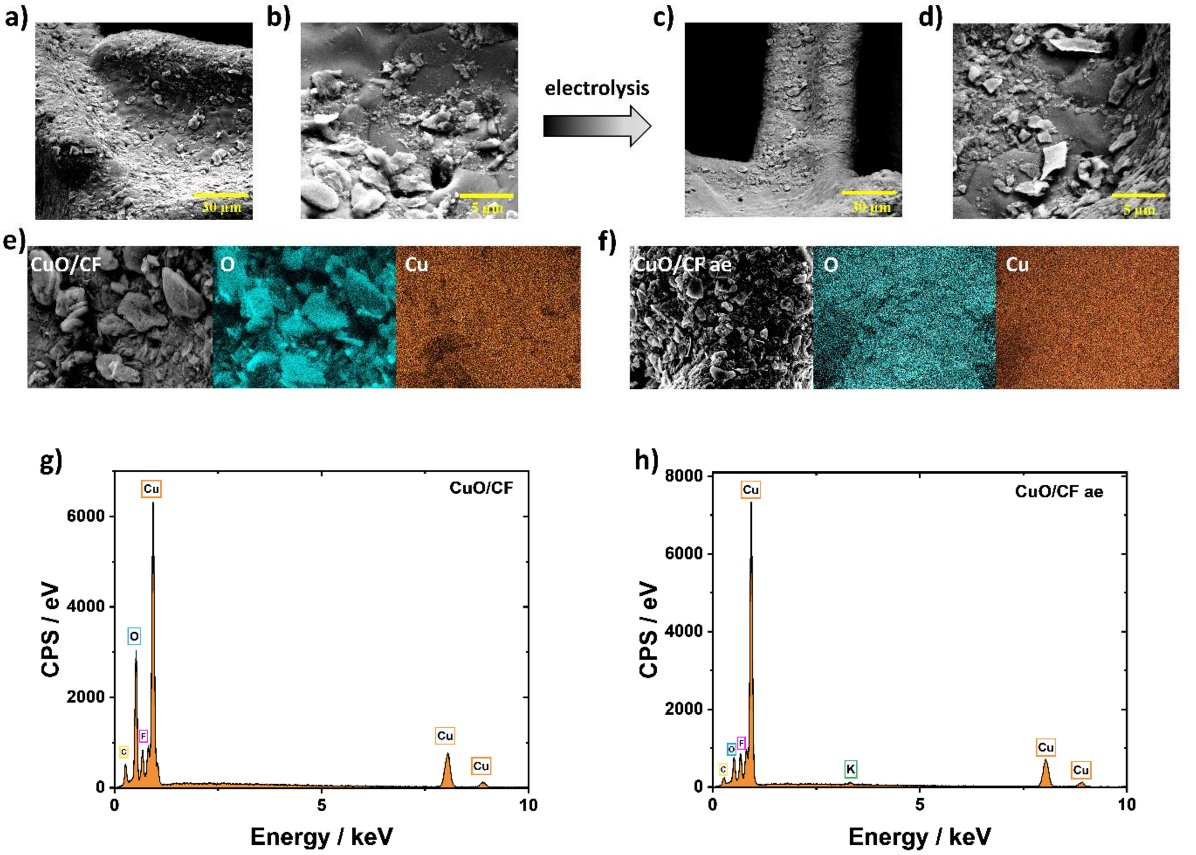

Supplementary Fig. 9: SEM-EDX measurements of CuO/CF before and after electrolysis. a) and b) SEM images of CuO/CF before electrolysis at 1000x and 5000x magnification. c) and d) SEM images of CuO/CF after electrolysis (ae) at 1000x and 5000x magnification. e) SEM EDX images before electrolysis of CuO/CF with O in torques and Cu in orange. f) SEM EDX images after electrolysis of CuO/CF with O in torques and Cu in orange. g) SEM EDX results before electrolysis of CuO/CF with O in torques, C in yellow, F in pink and Cu in orange. h) SEM EDX results after electrolysis of CuO/CF with O in torques, C in yellow, F in pink and Cu in orange.

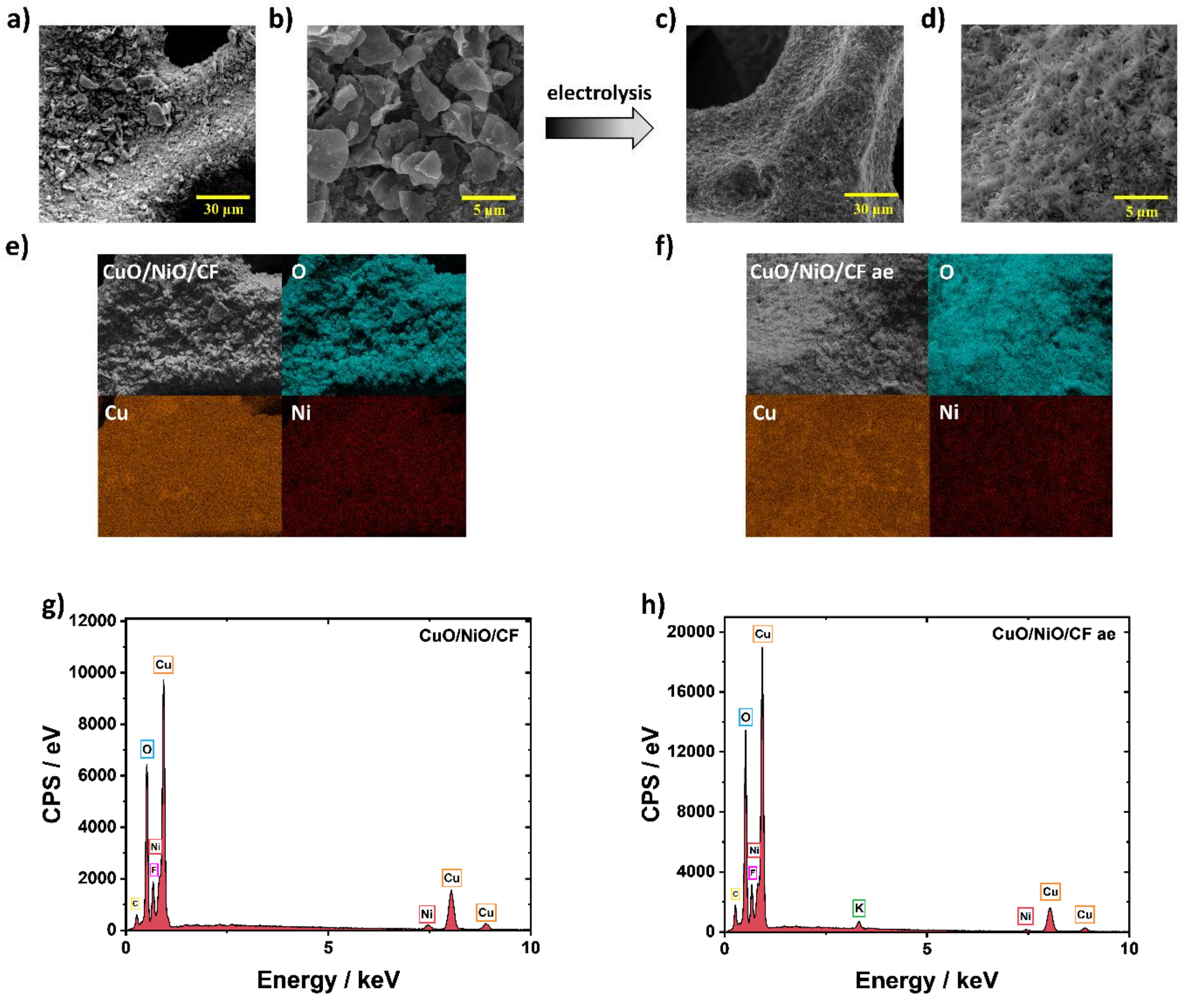

Supplementary Fig. 10: SEM-EDX measurements of CuO/NiO/CF before and after electrolysis. a) and b) SEM images of CuO/NiO/CF before electrolysis at 1000x and 5000x magnification. c) and d) SEM images of CuO/NiO/CF after electrolysis (ae) at 1000x and 5000x magnification. e) SEM EDX images before electrolysis of CuO/NiO/CF with O in torques, Ni in red and Cu in orange. f) SEM EDX images after electrolysis of CuO/NiO/CF with O in torques, Ni in red and Cu in orange. g) SEM EDX results before electrolysis of CuO/NiO/CF with O in torques, C in yellow, F in pink, Ni in red and Cu in orange. h) SEM EDX results after electrolysis of CuO/NiO/CF with O in torques, C in yellow, F in pink, Ni in red and Cu in orange.

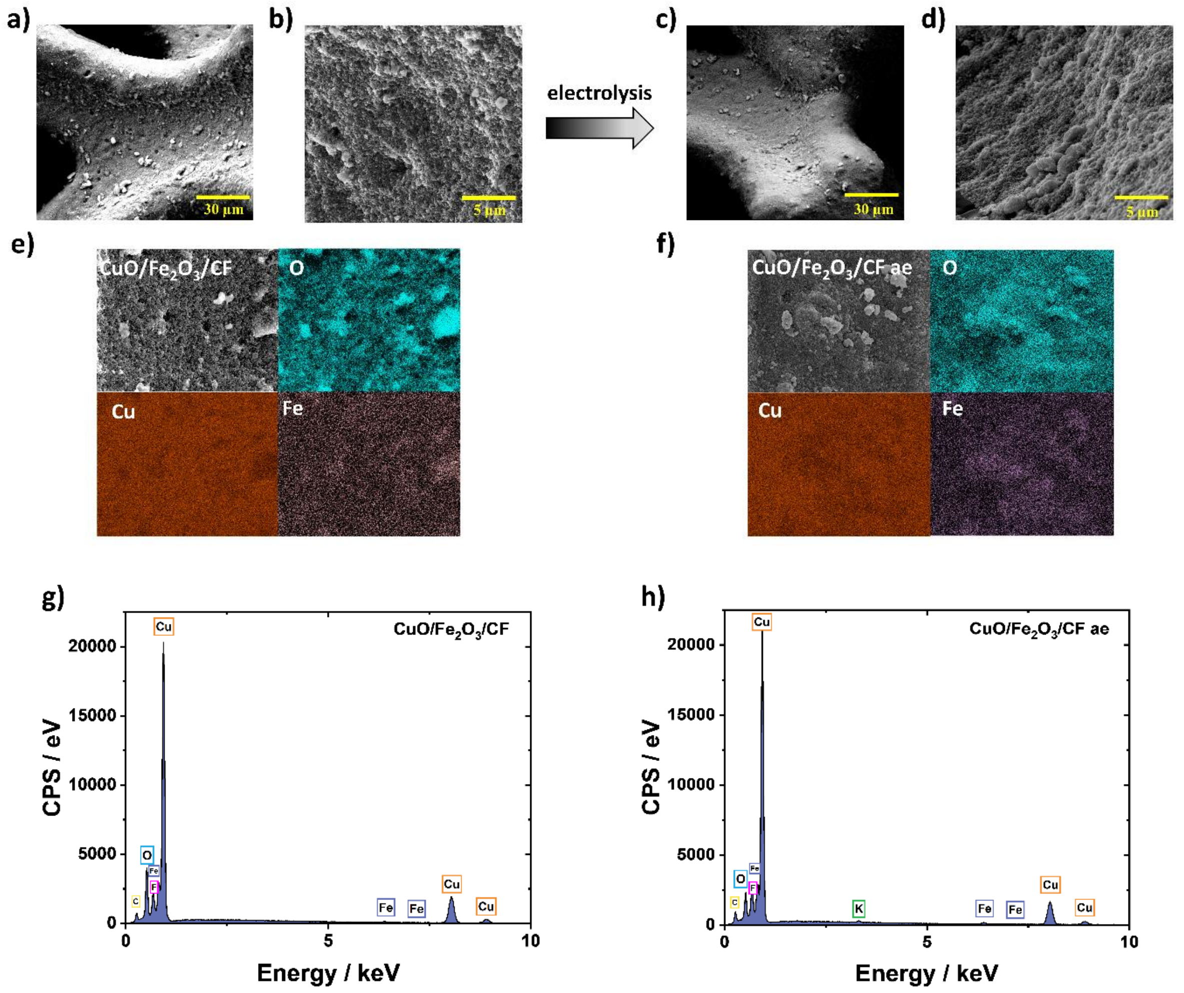

Supplementary Fig. 11: SEM-EDX measurements of $CuO/Fe_2O_3/CF$ before and after electrolysis. a) and b) SEM images of $CuO/Fe_2O_3/CF$ before electrolysis at 1000x and 5000x magnification. c) and d) SEM images of $CuO/Fe_2O_3/CF$ after electrolysis (ae) at 1000x and 5000x magnification. e) SEM EDX images before electrolysis of $CuO/Fe_2O_3/CF$ with O in torques, Fe in purple and Cu in orange. f) SEM EDX images after electrolysis of $CuO/Fe_2O_3/CF$ with O in torques, Fe in purple and Cu in orange. g) SEM EDX results before electrolysis of $CuO/Fe_2O_3/CF$ with O in torques, C in yellow, F in pink, Fe in purple and Cu in orange. h) SEM EDX results after electrolysis of $CuO/Fe_2O_3/CF$ with O in torques, C in yellow, F in pink, Fe in purple and Cu in orange.

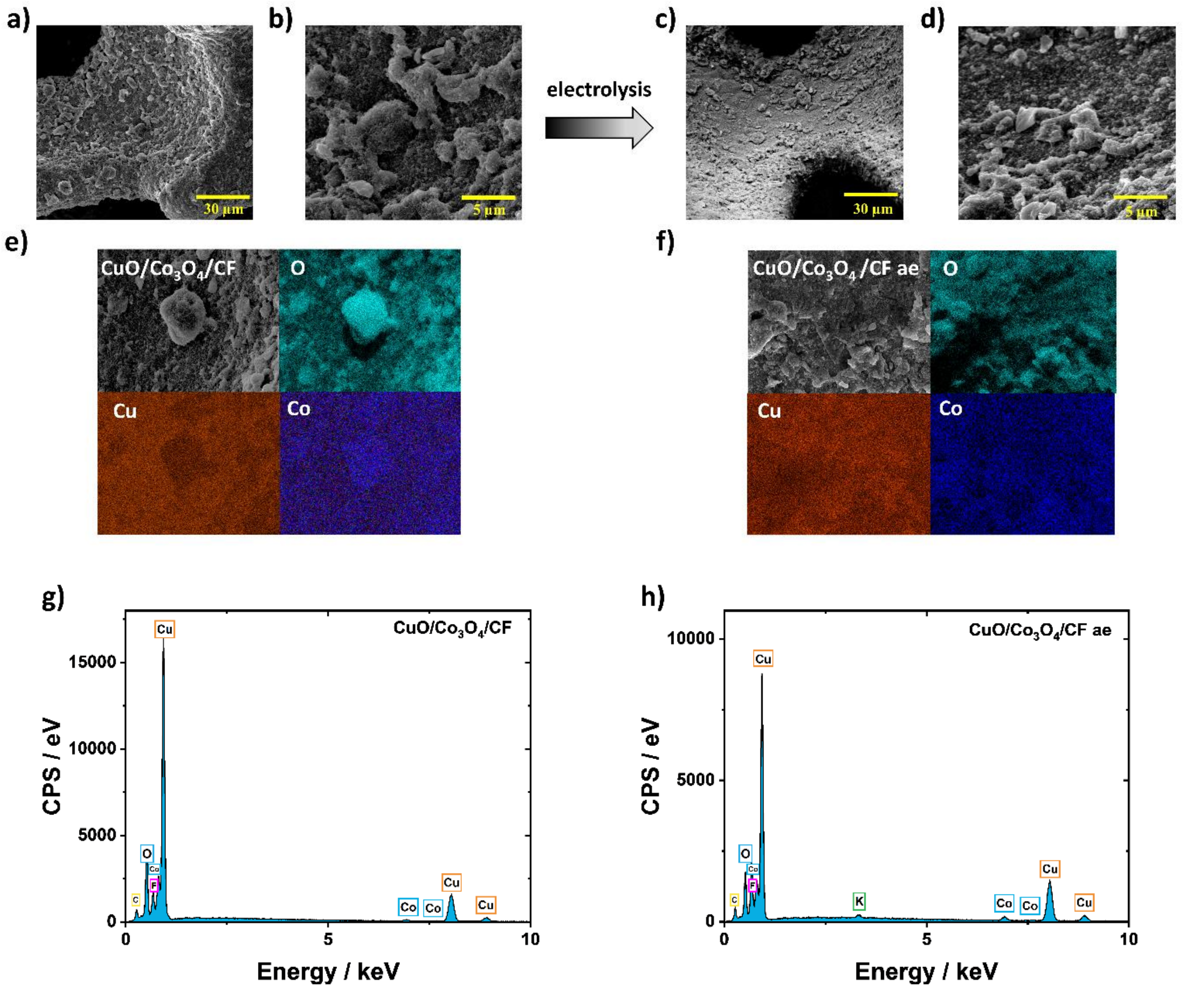

Supplementary Fig. 12: SEM-EDX measurements of $CuO/Co_3O_4/CF$ before and after electrolysis. a) and b) SEM images of $CuO/Co_3O_4/CF$ before electrolysis at 1000x and 5000x magnification. c) and d) SEM images of $CuO/Co_3O_4/CF$ after electrolysis (ae) at 1000x and 5000x magnification. e) SEM EDX images before electrolysis of $CuO/Co_3O_4/CF$ with O in torques, Co in blue and Cu in orange. f) SEM EDX images after electrolysis of $CuO/Fe_2O_3/CF$ with O in torques, Co in blue and Cu in orange. g) SEM EDX results before electrolysis of $CuO/Co_3O_4/CF$ with O in torques, C in yellow, F in pink, Co in blue and Cu in orange. h) SEM EDX results after electrolysis of $CuO/Co_3O_4/CF$ with O in torques, C in yellow, F in pink, Co in blue purple and Cu in orange.

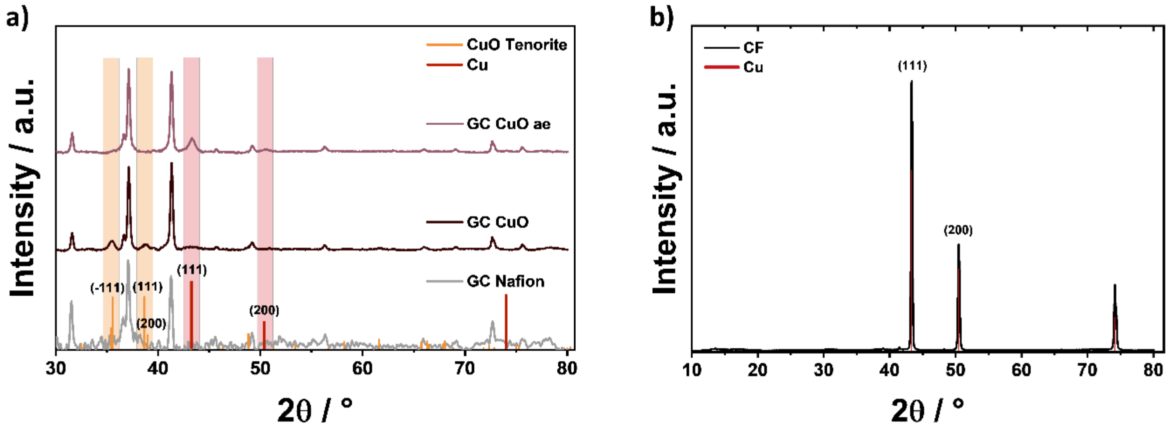

Supplementary Fig. 13: Thin film XRD of CuO on a GC disc electrode before and after electrolysis and of CF. a) XRD patterns of powder CuO drop casted on a GC disk electrode before (dark red) and after electrolysis (ae) (light red) and the GC disk electrode with Nafion binder as a background (grey), references are given in orange for CuO tenorite and red for metallic copper. The transparent boxes mark the specific 2θ angles for CuO tenorite (orange) and metallic copper (red). b) XRD patterns of CF (black) and the metallic copper reference (red)

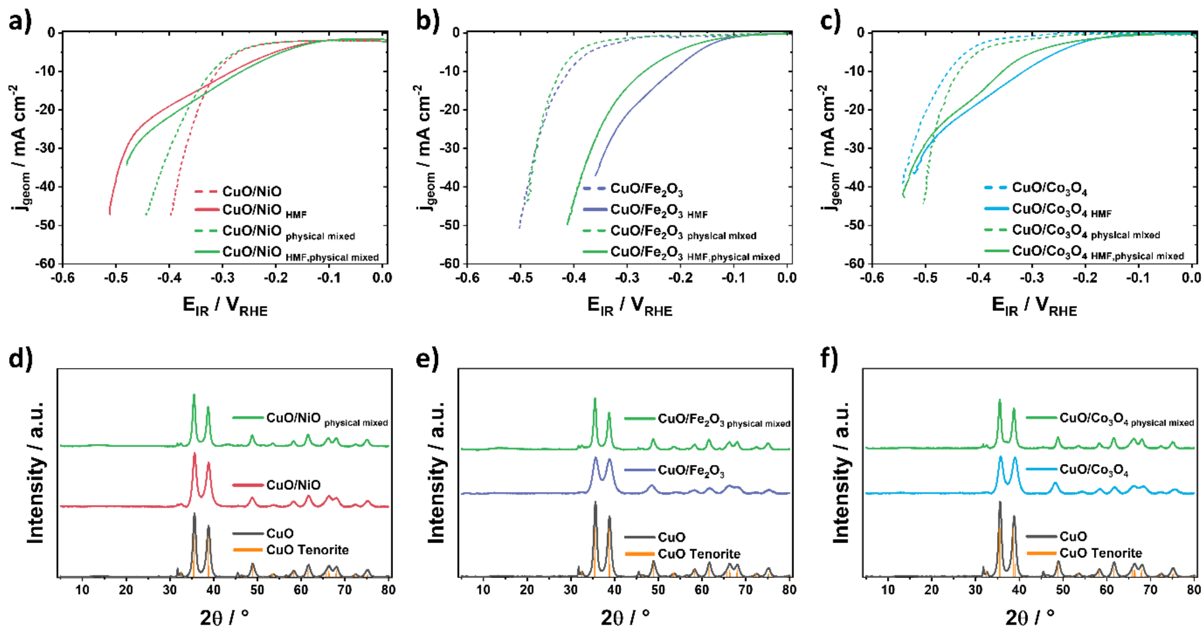

Supplementary Fig. 14: RDE three-electrode measurements and powder XRD characterization of CuO/MO$_x$ compared to physically mixed CuO and MO$_x$. a)-c) RDE measurements of the mixed metal oxides CuO/NiO (red), CuO/Fe_2O_3 (purple), and CuO/Co_3O_4 (blue) compared to the physically mixed equivalents (green). All RDE LSV measurements were taken between 0 V_{RHE} to -0.6 V_{RHE} at a scan rate of 10 mV s^{-1} in 0.1 M KOH with (solid line) and without (dashed line) 10 mM HMF at 2500 rpm with an electrode surface area of 0.19 cm^2 and a catalyst loading of 0.04 mg. All measurements are 100% manual internal resistance (IR) corrected. d)-f) Powder XRD measurements of the mixed metal oxides CuO/NiO (red), CuO/Fe_2O_3 (purple), and CuO/Co_3O_4 (blue) compared to the physically mixed equivalents (green), CuO (black), and the CuO Tenorite reference (orange).

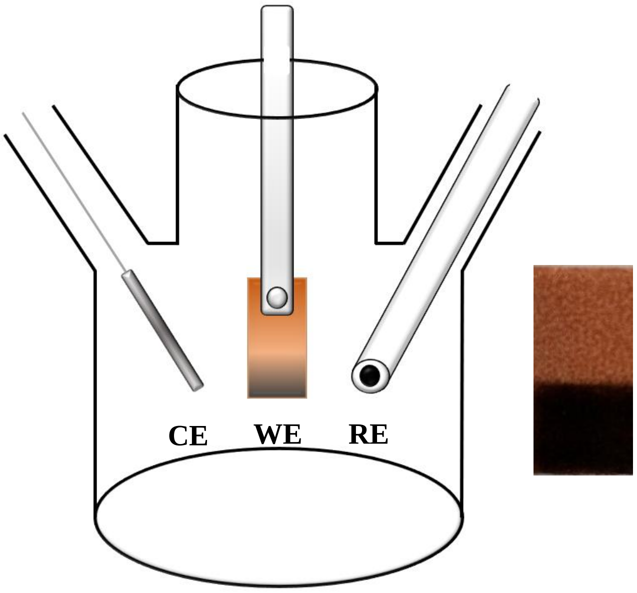

Supplementary Fig. 15: Schematic drawing of the undivided three-electrode cell setup. Showing the configuration of the UTEC from left to right, with Pt-mesh counter electrode (CE), 1 cm^{-2} CF based working electrode (WE) and reversible hydrogen electrode (RHE) reference electrode (RE) and an electrolyte volume of 50 ml.

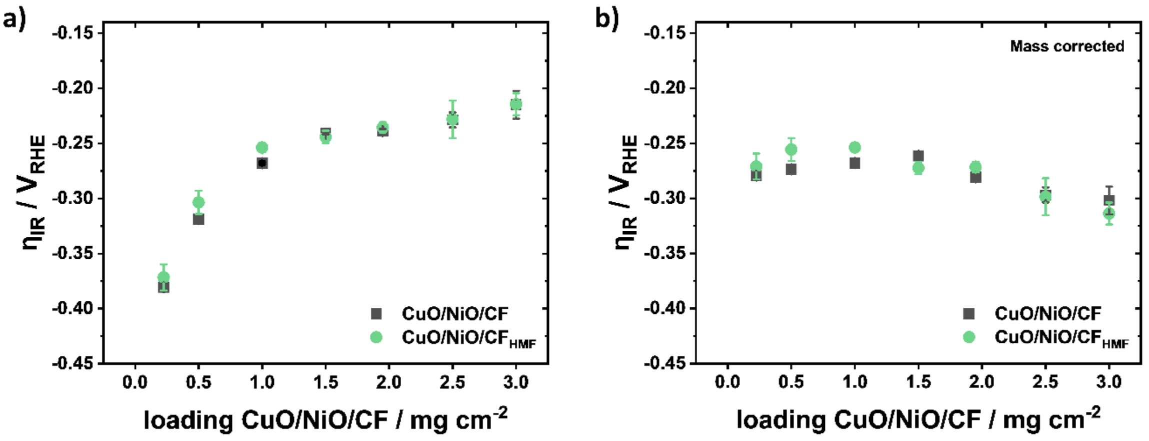

Supplementary Fig. 16: Undivided three-electrode cell (UTEC) loading study of CuO/NiO/CF. a) Showing the overpotential at 10 mA cm^{-2} for different catalyst loadings without (black) and with (green) 10 mM HMF. b) Showing the overpotential at 10 mA cm^{-2} (mass corrected) for different catalyst loadings without (black) and with (green) 10 mM HMF. The loading of 0 mg cm^{-2} is out of the plotted range.

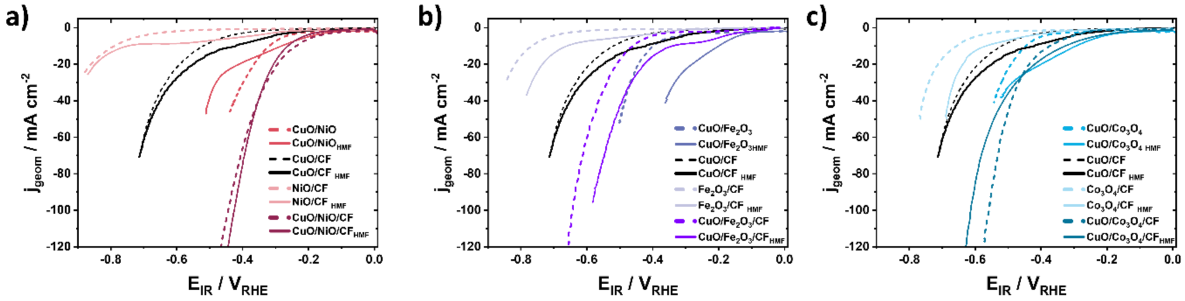

Supplementary Fig. 17: Activity comparison of CuO/MOx, CuO/CF, MOx/CF, and CuO/MOx/CF. Comparing a) CuO/NiO (red), CuO/CF (black), NiO/CF (light red), and CuO/NiO/CF (dark red), b) CuO/Fe_2O_3 (purple), CuO/CF (black), Fe_2O_3/CF (light purple) and CuO/ Fe_2O_3/CF (shiny purple) and c) CuO/Co_3O_4 (blue), CuO/CF (black), Co_3O_4/CF (light blue) and CuO/ Co_3O_4/CF (dark blue). Reaction conditions are the same as in Figure 3 for RDE and UTEC measurements. All measurements are 100% manual internal resistance (IR) corrected.

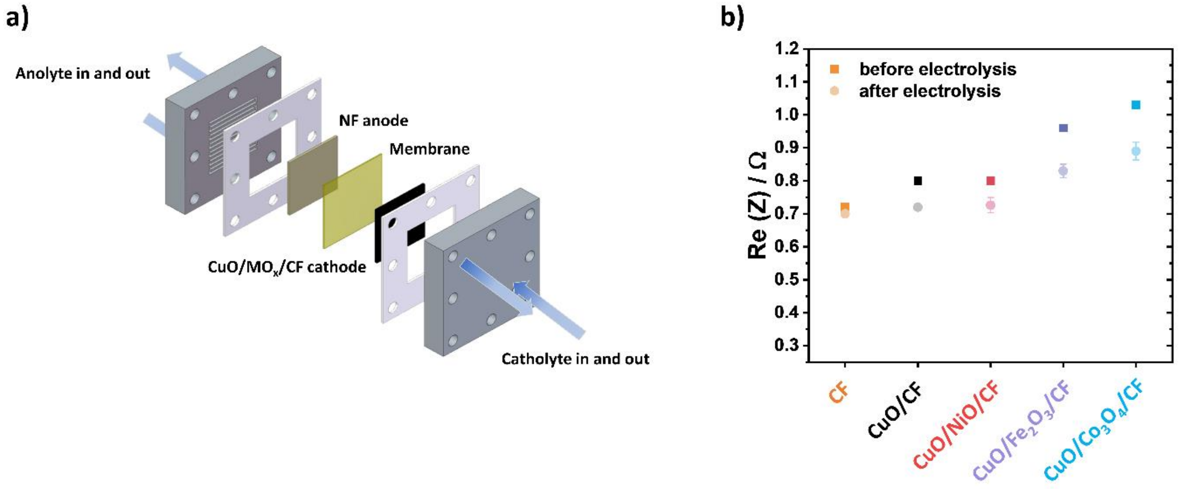

Supplementary Fig. 18: Electrochemical cell setup and the corresponding HFR results. a) 0.1 M KOH with 10 mM HMF as catholyte (100 ml, recycled), 0.1 M KOH as anolyte (100 ml, recycled), 5 cm^2 electrode area, CuO/MOx/CF as cathode (black), nickel foam (NF) as anode (metallic grey), FAA-3-PK membrane (yellow), PTFE gaskets (white) and a flow rate of 25 ml min^{-1}. Based on the same design from earlier work.[1] b) High frequency resistance results before and after constant current electrolysis. Error bars with relative errors of 0.4-2%.

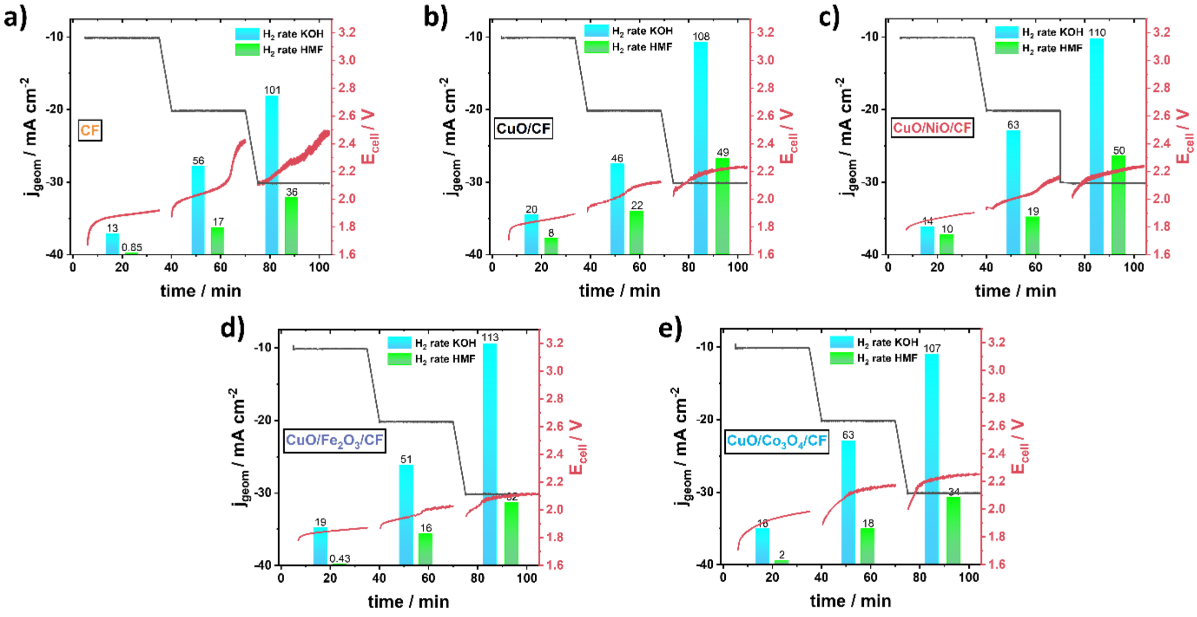

Supplementary Fig. 19: MEA-Flow-Cell activity measurements of CuO/MO$_x$/CF electrodes. a)-e) Cell potential (red), current densities (black) and H_2 rate without (neon blue) and with HMF (neon green) of the spray-coated CuO/MO$_x$/CF catalysts pure CF (orange), CuO (black), NiO (red), Fe_2O_3 (purple) and Co_3O_4 (blue). Cell reaction conditions: 0.1 M KOH without 10 mM HMF as anolyte (100 ml), and 0.1 M KOH with 10 mM HMF as catholyte (100 ml), 5 cm^2 electrode area, nickel foam (NF) as anode and a flow rate of 25 ml min^{-1}, at 10-30 mA cm^{-2}. High frequency resistance results are between 0.7- 1.05 Ω (Supplementary Fig18b).

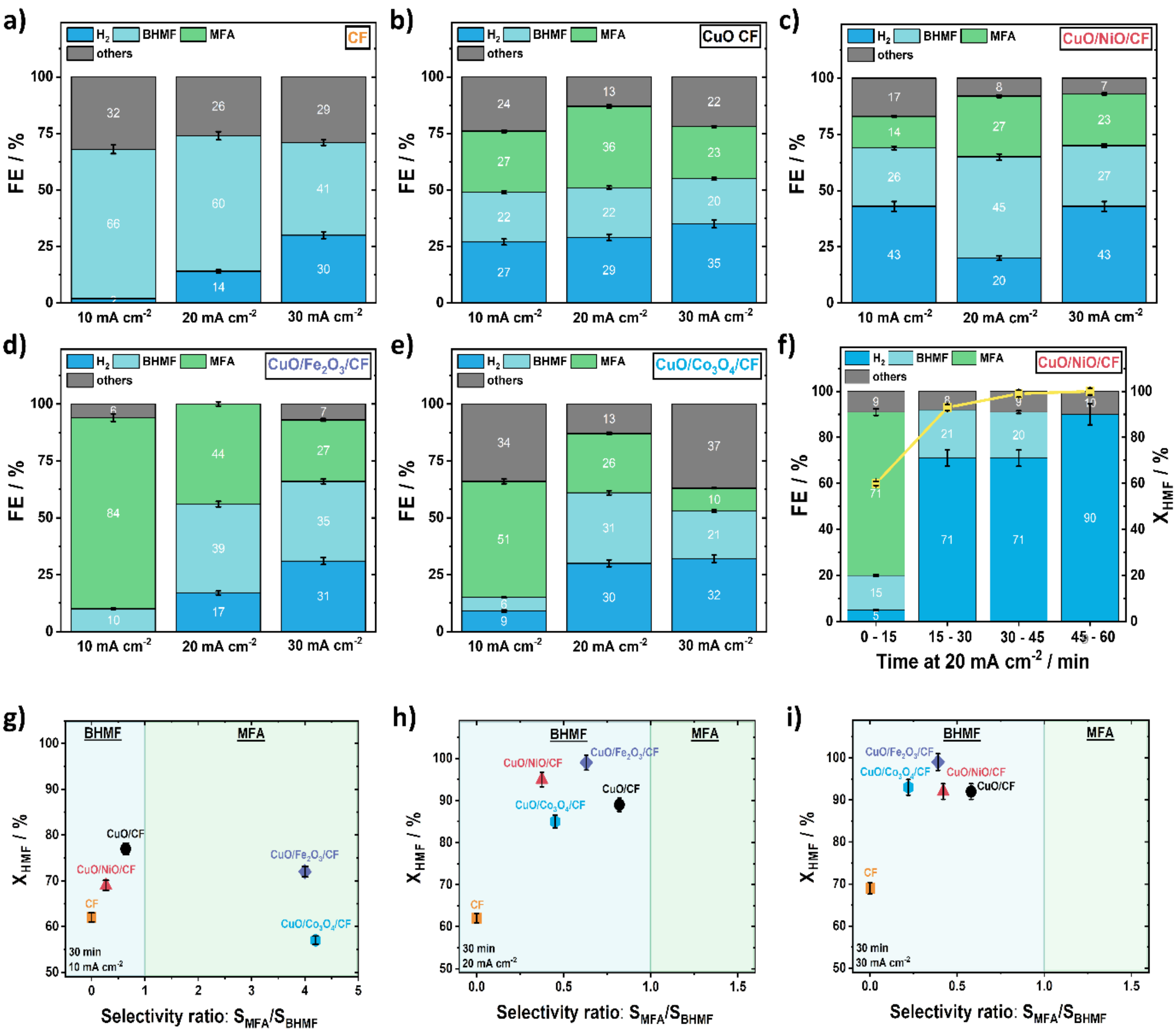

Supplementary Fig. 20: MEA-Flow-Cell performance measurements of CuO/MO$_x$/CF electrodes (with error bars). a)-e) Faradaic efficiencies for H_2 (strong blue), BHMF (light blue), MFA (green) and other products (grey) of the different spray-coated CuO/MO$_x$/CF catalysts. f) Faradaic efficiencies and HMF conversion (yellow) are calculated for every 15 min time interval over 60 min using CuO/NiO/CF as a catalyst. Product color code stays as in a)-e). g)-i) Scatter plot of the product selectivity preference. HMF conversion over MFA/BHMF selectivity ratio, calculated by S_{MFA}/S_{BHMF} for all CuO/MO$_x$ catalysts on CF at different current densities for 30 min. The color code stays the same as before. Cell reaction conditions: 0.1 M KOH with 10 mM HMF as catholyte (100 ml, recycled), 0.1 M KOH as anolyte (100 ml, recycled), 5 cm^2 electrode area, nickel foam (NF) as anode and a flow rate of 25 ml min^{-1}, at 10-30 mA cm^{-2} for 30 min. High frequency resistance results are between 0.7- 1.05 Ω (Supplementary Fig18b). Error bars with relative errors of 2-4% for $FE_{products}$, 3-5% for FE_{H2} and 1-3% for X_{HMF} were added.

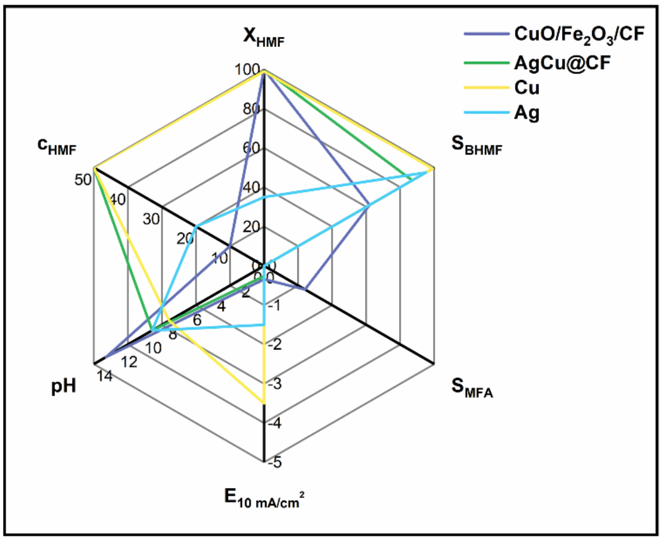

Supplementary Fig. 21: Comparison with the literature presented in a radar plot. Performance and reaction parameters like the HMF conversion (X_{HMF}), selectivity towards BHMF (S_{BHMF}) and MFA (S_{MFA}), the potential at 10 mA cm^{-2} ($E_{10mA/cm2}$), pH and initial HMF concentration (c_{HMF}) are compared between CuO/Fe_2O_3/CF and data from the literatur.[2-4]

Supplementary Table 1: ICP-OES results of the bimetallic catalysts. The composition of powdered CuO/NiO, CuO/Fe_2O_3 and CuO/Co_3O_4 are given in mol% and wt%.

**Catalyst CuO/ MO_x**	**M/Cu mol%**	**Cu wt%**
CuO/NiO	10.26	54.32
CuO/Fe_2O_3	10.29	51.12
CuO/Co_3O_4	10.72	55.20

Supplementary Table 2: BET results in m^2/g and in cm^2/0.04 mg (RDE electrode catalyst loading).

**Surface Area**	**CuO com.**	**CuO**	**NiO**	**Fe_2O_3**	**Co_3O_4**	**CuO/NiO 10 mol%**	**CuO/Fe_2O_3 10 mol%**	**CuO/Co_3O_4 10 mol%**
**[m^2/g]**	11.06	21.78	183.89	95.98	114.79	68.54	84.20	87.97
**[cm^2/0.04mg]**	0.04	0.08	0.73	0.38	0.45	0.27	0.33	0.35

Supplementary Table 3: Performance parameters of all catalysts at different current densities over 30 min in the MEA flow cell.

Catalyst	j [mA cm^{-2}]	X_{HMF} [%]	Y_{BHMF} [%]	S_{BHMF} [%]	FE_{BHMF} [%]	Y_{MFF} [%]	S_{MFF} [%]	FE_{MFF} [%]	Y_{MFA} [%]	S_{MFA} [%]	FE_{MFA} [%]	H_2 rate	FE_{H2} [%]
CF	10	62	31	50	66	-	-	-	-	-	-	0.85	2
	20	62	56	90	60	-	-	-	-	-	-	16.73	14
	30	69	57	83	41	-	-	-	-	-	-	35.76	30
CuO/CF	10	77	10	14	22	-	-	-	7	9	27	8.25	27
	20	89	20	23	22	1	1	2	17	19	36	22.33	29
	30	92	29	31	20	1	1	1	17	18	23	48.63	35
CuO/NiO/CF	10	69	12	18	26	-	-	-	4	5	14	9.9	43
	20	95	46	48	49	-	-	-	17	18	30	18.61	22
	30	92	40	43	27	-	-	-	17	18	23	50.01	43
CuO/Fe_2O_3/CF	10	72	5	7	10	-	-	-	20	28	84	0.43	0
	20	99	38	38	39	-	-	-	24	24	44	16.33	18
	30	99	61	62	35	-	-	-	24	24	27	32.33	31
CuO/Co_3O_4/CF	10	57	3	5	7	-	-	-	12	21	51	1.45	9
	20	85	26	31	28	-	-	-	12	14	26	17.8	30
	30	93	30	32	21	-	-	-	7	7	10	34.09	32
CuO/Fe_2O_3/CF NiFe(-Cl^-)-LDH@NF	20	99	40	40	40	-	-	-	28	28	57		

Supplementary References

1 Hauke, P., Klingenhof, M., Wang, X., de Araújo, J. F. & Strasser, P. Efficient electrolysis of 5-hydroxymethylfurfural to the biopolymer-precursor furandicarboxylic acid in a zero-gap MEA-type electrolyzer. *Cell Reports Physical Science* **2**, 100650, doi:https://doi.org/10.1016/j.xcrp.2021.100650 (2021).

2 Sanghez de Luna, G. *et al.* AgCu Bimetallic Electrocatalysts for the Reduction of Biomass-Derived Compounds. *ACS Applied Materials & Interfaces* **13**, 23675-23688, doi:10.1021/acsami.1c02896 (2021).

3 Zhang, Z. *et al.* Operando Generated Copper-based Catalyst Enabling Efficient Electrosynthesis of 2,5-Bis(hydroxymethyl)furan. *Fundamental Research*, doi:https://doi.org/10.1016/j.fmre.2022.01.016 (2022).

4 Liu, H., Lee, T.-H., Chen, Y., Cochran, E. W. & Li, W. Paired electrolysis of 5-(hydroxymethyl)furfural in flow cells with a high-performance oxide-derived silver cathode. *Green Chemistry* **23**, 5056-5063, doi:10.1039/D1GC00988E (2021).
